# Adverse childhood experiences and multimorbidity of internalising and cardiometabolic conditions in an older-age population.

**DOI:** 10.23889/ijpds.v9i5.2618

**Published:** 2024-09-18

**Authors:** Mikayla Hunter, Nathan Nickel, LINC Consortium

**Affiliations:** 1 University of Manitoba

Information regarding biological sex is collected for administrative and clinical purposes, and often is conflated with gender in clinical encounters as well as in population-based data studies. Sex is collected as either ‘male’ or ‘female’, adhering to the colonial notion of binary sex and gender identities. Meaningful information on sexual/romantic orientation is also absent from these datasets. Sex, gender, and sexual/romantic (SR) orientation are key aspects informing how people experience the world and how we interpret administrative data.

SOGI-HE (Sexual Orientation and Gender Identity – Health Equity) will be the first 2SLGBTQIA+ dataset at the Manitoba Centre for Health Policy by using a novel survey tool to collect information on gender identity, and SR orientation. The data from this survey will be linked into the Population Health Data Repository housed at the Manitoba Centre for Health Policy, enabling analyses that incorporate constructs of sex, gender, and SR orientation. These data will make visible the experiences of the 2SLGBTQIA+ community that have been historically absent from population-based data studies. Data equity and 2SLGBTQIA+ visibility within data play an important role in advancing the field and lays the groundwork for future data linkage projects.

In this presentation we will discuss the development of SOGI-HE through community-based research principles and the implications it has on future 2SLGBTQIA+ research conducted in the province of Manitoba (Canada). Further, we will discuss the ethical implications associated with queer data sovereignty in a precarious political climate for 2SLGBTQIA+ people and their rights.

